# *Moringa oleifera* Seed at the Interface of Food and Medicine: Effect of Extracts on Some Reproductive Parameters, Hepatic and Renal Histology

**DOI:** 10.3389/fphar.2022.816498

**Published:** 2022-03-08

**Authors:** Alfred F. Attah, Opeyemi O. Akindele, Petra O. Nnamani, Ugochukwu J. Jonah, Mubo A. Sonibare, Jones O. Moody

**Affiliations:** ^1^ Department of Pharmacognosy, Faculty of Pharmacy, University of Ibadan, Ibadan, Nigeria; ^2^ Department of Pharmacognosy and Drug Development, Faculty of Pharmaceutical Sciences, University of Ilorin, Ilorin, Nigeria; ^3^ Laboratory for Reproductive Physiology and Developmental Programming, Department of Physiology, College of Medicine, University of Ibadan, Ibadan, Nigeria; ^4^ Drug Delivery and Nanomedicines Research Unit/Public Health and Environmental Sustainability (PHES) Research Group, Department of Pharmaceutics, University of Nigeria, Nsukka, Nigeria; ^5^ Riyadh Al Khabra General Hospital, Riyadh, Saudi Arabia

**Keywords:** *Moringa oleifera*, seed, Wistar rats, hepatic toxicity, renal histology, reproductive parameters, developmental programming

## Abstract

The lipid-rich Seed of *Moringa oleifera* has been promoted as an effective water clarifier. Aside its vital nutritional application as an emerging food additive, the seed has continued to gain a wider acceptance in various global ethnomedicines for managing several communicable and lifestyle diseases, howbeit, its potential toxic effect, particularly on fertility and pregnancy outcomes has remained uninvestigated; the effect of *Moringa oleifera* seed (MOSE) aqueous-methanol extracts on fertility and pregnancy outcome, was investigated *in vivo* using female Wistar rats that were divided into 50, 100, 300 and 500 mg per kilogram body weight. Group six was given *Moringa oleifera* seed treated water *ad-libitum* (*ad-libitum* group). Organs harvested for histological assessment included ovary, uterus, liver and kidney. In addition to HPLC fingerprint and a preliminary peptide detection, we determined the physico-chemical characteristics and mineral content of MOSE using standard methods. Data were analyzed with significance at *p* ≤ 0.05. There was no significant difference in the estrus cycle, mating index, gestation survival index, gestation index, fertility index and sex ratio among all groups. Gestation length was reduced in some groups. While the male pup birth weight was comparable among the different groups, female pups birth weights were significantly reduced in 50 and 100 mg groups. Anogenital distance indices of female pups in *ad libitum* group were significantly increased. Pathologies were observed in liver and kidneys of dams while kidneys of pups presented a dose dependent reduction in the number of glomeruli. There were no observed pathological changes in the ovary and uterus. This study showed for the first time in rodents, that the lipid-rich MOSE is unsafe to the kidney of rodents while the lipid-free MOSE appears to be safe at doses up to 300 mg/kg body weight. Findings from this study suggested that the female pups were masculinized. In conclusion, the lipid-rich seed extracts of MOSE appear to be unsafe during pregnancy, induce hepatic and renal toxicity while the lipid-free MOSE excludes inherent toxicity as the hydrophobic part has been linked to toxicity as observed in this study due to the developmental programming effect on female offspring in rodents.

## 1 Introduction

Based on accumulating scientific evidence, increasing number of plants are gaining acceptance as important sources of nutrients, required for growth, development, and prevention of lifestyle diseases (Witkamp and van Norren, 2018; Cisneros-Zevallos, 2021; Marques et al., 2021). Plants constitute an amazingly rich source of secondary metabolites, which have served as templates or active molecules for the prevention, treatment or cure for several diseases threatening mankind (Ashwin et al., 2021; Leuci et al., 2021; Pizzi, 2021). The application of edible plant species as both food and medicine has been well documented in countries endowed with rich Traditional Medicine and plant biodiversity (Cisneros-Zevallos, 2021; de Medeiros et al., 2021). Interestingly, the advancement in science, improved understanding of the crucial role of nutrition or more generally, lifestyle, in disease prevention/management tend to lend support to the practice of utilizing wild and cultivated botanicals at the interface of food and medicine. Thus, the popular phrase “Let thy food be thy medicine and thy medicine thy food” is increasingly gaining relevance in the 21st century (Witkamp and van Norren, 2018). *Moringa oleifera* (MO) Lam. (Moringaceae) and related 12 species within the genus Moringa is a typical example representing one of the most valued and widely used ethnomedicinal plant species occupying the food-medicine interface (Palada, 2019; Jattan et al., 2021).

*Moringa oleifera* (MO) is a resilient tree cultivated mainly within the tropical and sub-tropical regions of the world whose origin has been linked to two continents of Asia and Africa (Lakshmidevamma et al., 2021). More striking is the oral histories recorded by Lowell Fuglie in Senegal and many other parts of West Africa where the lifesaving nutritional applications of MO (Fuglie, 1999; Fahey, 2005) has continued to be harnessed. The mature and dry seeds from MO have been found useful in several traditional cultures especially those at the base of the economic ladder who lack access to clean drinking water. These native people have historically been applying crushed seeds obtained from MO to effect the clarification of turbid water for household use. The traditional technology was frequently observed with herdsmen whose main sources of drinking water have been muddy and cloudy lakes or running water. Thus, the use of crushed *Moringa oleifera* seeds (MOSE) as a cheap, accessible, affordable, sustainable and acceptable alternative for the treatment of unsafe water from multiple sources is well documented and scientifically validated (Gassenschmidt et al., 1995; Ghebremichael et al., 2005). Moringa seed is rich in lipids, with over 35% of each kernel containing mainly unsaturated fatty acids and other lipophilic metabolites (Ayerza, 2012), it is therefore logical to assume that crushed seeds locally applied to clarify polluted water will inadvertently leave some suspended oils and related metabolites in the treated water. It is reported in the literature that flocculant phytochemicals released from the seeds coagulate 80.0–99.5% turbidity with a concomitant bacterial load reduction of 90.0–99.99% (Madsen et al., 1987; Ueda Yamaguchi et al., 2021). Polypeptides present in the seeds have been implicated as the main flocculant molecules (Ghebremichael et al., 2005). Other secondary compounds isolated from the seed are lipids and glycosides including *O*-ethyl-4-(α-L-rhamnosyloxy) benzyl carbamate, 4(α-L-rhamnosyloxy)-benzyl isothiocyanate, niazimicin, 3-*O*-(6′-*O*-oleoyl-β-D-glucopyranosyl)-β-sitosterol, β-sitosterol-3-*O*-β-D-glucopyranoside, niazirin, β-sitosterol and glycerol-1-(9-octadecanoate) (Guevara et al., 1999; Dzuvor et al., 2022). Some of the Moringa fatty acids and lipids have been reported as phytochemical agents with uterotonic potential (Gruber and O'Brien, 2011) making their intake potentially unsafe during pregnancy.

Many phytochemicals (chemical compounds of botanical origin) including steroidal alkaloids, pyridine-pyrrolidine alkaloids, substituted phenolic compounds, steroidal and triterpenoid glycosides, have been reported to affect growth and reproduction in animals (Francis et al., 2002). For instance, nicotinic alkaloids have been reported to produce morphological and behavioral changes in rodents (Khalki et al., 2012). Robert and colleagues (Kavlock et al., 1991) reported the *in vitro* developmental toxicity of phenolic compounds and demonstrated that once a phenol successfully gains entry into the embryo, the embryotoxicity of the compound is dependent on the degree of its lipophilicity. In other words, hydrophobic phenols appear to be more toxic to the developing embryo than highly polar phenols (Selassie et al., 1999). Using an effective concentration of less than 0.5 mM, the group reported the effect of 13 phenolic compounds in producing growth retardation and structural abnormalities *in vitro*. However, the findings from *in vivo* investigation suggest that only few of these substituted phenols, the lipophilic type actually produced statistically relevant developmental toxicity. The investigators used a dose range of 0–1000 mg/kg body weight of rodents. Furthermore, the potential *in vivo* embryotoxicity and teratogenicity of some botanical terpenes has also been reported; employing the embryonic *Danio rerio* (family: Cyprinidae) model, the teratogenic potentials of terpenes has been demonstrated following documented morphological defects, decreased hatchability and reduced heart rate (Thitinarongwate et al., 2021).

Raising scientific curiosity for further *in vivo*-inspired investigation is the report by Al-Anizi et al. (2014) regarding the cytotoxic and genotoxic potentials of the hydrophobic fraction (basically lipids) of MOSE. Meanwhile, folkloric claims regarding the abortifacient potential of some aerial parts of *Moringa oleifera* plant have been documented and scientifically validated (Nath et al., 1992; Fahey, 2005; Attah et al., 2020) and this should inform an elaborate *in vivo* reproductive toxicity studies on the seed of the plant which has now occupied an important global position at the interphase of food and medicine. However, until now, there are currently no reported scientific findings on the potential effects of MOSE on fertility and pregnancy outcome. Yet the application of lipid-rich Moringa whole seed as well as the aqueous extracts of MOSE (containing suspended lipids) as an anti-infective and restorative nutraceutical (Xiong et al., 2020; Mohanty et al., 2021; Oyeleye et al., 2021; Reyes-Becerril et al., 2021; Santos et al., 2021; Xiong et al., 2021), as food additive (Milla et al., 2021; Okereke et al., 2021; Oyeleye et al., 2021) as well as for polluted water treatment in deprived rural settings (Lea, 2010; Palada, 2019; Aboagye et al., 2021; Ueda Yamaguchi et al., 2021) has continued to gain momentum across different regions of the world without any attention to its potential toxicities. Here, the effects of the lipid-containing MOSE aqueous-methanol extracts on fertility and pregnancy outcome have been elaborately investigated *in vivo* using rodents for their potential effects on reproductive parameters, ovary, uterus, renal and hepatic histology. Furthermore, the potential safety of the extract of the lipid-free seedcake as well as its physicochemical characteristics have been documented.

## 2 Materials and Methods

### 2.1 Plant Materials and Extract Preparation

Approximately 3 kg of *Moringa oleifera* seeds freshly harvested from organic farmland in Zaria, Nigeria were obtained from Herbal Point, a local herbal medicinal store and the main distributor of MO products located in Zaria, Kaduna State, Nigeria. Viable seeds were further cultivated in a household plant garden in Ibadan, Nigeria and at least 3-months old growing plant samples were collected fresh and together with the seeds submitted for authentication at Forest Herbarium Ibadan (FHI). Voucher number was given (FHI: 109853) while the voucher specimen was deposited in the same herbarium. Further, seeds were mechanically hulled to remove the hulls in order to obtain the clean whitish seed kernels. To obtain the lipid-containing aqueous-methanol extract, the powdered material was extracted in distilled water/methanol (1:1 v/v) (Attah et al., 2016) for 24 h following occasional agitation. Freeze-dried and crispy samples were refrigerated until required for use. In preparing the lipid-free fraction, the lipid content was first removed using hexane. Briefly, the finely powdered kernels was extracted in food grade N-hexane for 24 h with occasional shaking at a sample/solvent ratio of 1:10 (w/v); filtration was carried out to separate out the marc (representing the seedcake). The well dried and crispy marc was further extracted with distilled water/methanol as described above. The freeze-dried aqueous extracts were kept refrigerated for the single dose 300 mg/kg body weight administration of the lipid-free MOSE. In addition, ∼100 mg of powdered unextracted whole seed kernel (representing one seed kernel) was used to treat 1 L of well water sourced from a neighboring rural community at the University of Ibadan, Nigeria. The duration of 30 min treatment time was considered. The resultant filtrate represents the Moringa treated water which has been prepared according to its indigenous application in various African households. This was administered to the *ad libitum* group that had unlimited free access to the treated water.

### 2.2 Experimental Animals

Mature female Wistar rats weighing between 250–300 g and 150–170 g were respectively used for this *in vivo* studies. Animals were fed with a standard rat diet (Ladokun feeds, Ibadan, Nigeria) and allowed free access to water *ad libitum*. Distilled water was used to reconstitute freeze dried extract prior to administration. Administration of all extracts used has been achieved via the oral route (oral gavage) once daily using a maximum volume of 2 ml, administered at 1 ml at a time. All procedures used in this study conformed to the guideline of the care and use of animals in research and teaching (NIH publication, revised 1996) and were in line with the University of Ibadan Animal Welfare and Ethics Committee guidelines. All animals were therefore handled humanely in line with the ethics committee guidelines (approval number: FVM/UI/A.6/201301).

### 2.3 *In-Vivo* Experimental Procedure

Thirty female Wistar rats were randomly divided into six groups and treated with *Moringa oleifera* seed extract (MOSE) as follows: Group one received 1 ml of distilled water per kilogram body weight (1 ml/kg body weight). Groups two, three, four, and five received 50, 100, 300 and 500 mg of MOSE extract per kilogram body weight. Group six was given *Moringa oleifera* seed treated water *ad-libitum* constituting the *ad-libitum* group.

### 2.4 Determination of Estrous Cycle Pattern

Estrous cycle pattern was studied for 4 weeks; 2 weeks prior to the commencement of the administration of MOSE (pre-treatment period) and 2 weeks during which MOSE was administered before the animals were paired for mating (post-treatment period). The estrous cycle was monitored during the pre-treatment period to establish the estrous pattern of each animal. Briefly, vaginal smears were obtained using sterile cotton-tipped swab wetted with physiological saline under an ambient temperature. This was introduced about 1 cm deep into the vagina orifice ensuring the lowest possibility for excessive cervical stimulation. The swab was quickly but carefully turned and rolled once against the wall of the vagina and then gently removed. Collected epithelial cells of the vaginal wall were further transferred to a clean dry glass slide by moving the swab across the slide. The slide was air-dried, stained, and viewed under the light microscope. Morphometric analysis of stained vaginal smears was carried out under a light microscope followed by the determination of the number of epithelial cell types in each phase of the estrous cycle; where one estrous cycle has been defined as the number of days from one estrus to the next estrus. Analyzed cell types within the vaginal secretion include leucocytes, cornified epithelial cells and nucleated epithelial cells. The phase of estrous cycle during the experiment was based on the proportion of these cells detected in the vaginal secretion (Marcondes et al., 2002; Ajayi and Akhigbe, 2020).

### 2.5 Mating Procedure

The animals were paired with proven male breeders at ratio 2:1 (female: male). Vaginal lavage of the paired female animal was examined every morning after mating using the microscope to determine the presence of sperm cells. The day spermatozoa were seen in the vagina smear of the rat was designated as day one of pregnancy. All female rats spent 14 days with the male rats. The animals were allowed to litter naturally and the day of parturition was designated as postnatal day one. In summary, MOSE was administered to the experimental animals for a maximum period of 35 days, starting from the post treatment period till parturition. Animals were weighed weekly throughout the experiment. Anogenital distance and other morphometric indices of the pups were measured within 24 h after parturition using a digital Vernier caliper (Donguan Hust Tony Instrument, China). Mating index, fertility index, gestation survival index, gestation index, gestation length, litter size and sex ratio were evaluated using the following formulae;

$$Mating\;index=\frac{Number\;of\;females\;mated}{Number\;of\;females\;cohabited}\times 100$$

$$\mathrm{Pr}egnancy\;index=\frac{Number\;of\;females\;delivering\;live\;newborn}{Number\;of\;females\;with\;evidence\;of\;pregnancy}\times 100$$

$$Delivery\;index=\frac{Number\;of\;delivering\;females}{Number\;of\;pregnant\;females}\times 100$$

$$Life\;birth\;index=\frac{Number\;of\;offspring\;living}{Number\;of\;offspring\;delivered}\times 100$$

### 2.6 Sacrifice

All pups were sacrificed on postnatal day 14 while the dams were sacrificed during their first proestrus after the sacrifice of their pups. All sacrifice was done by cervical dislocation. The kidneys, liver, ovaries and uteri were carefully harvested from the dams while only the kidneys and liver were harvested from the pups. Organs were fixed in 10% formal saline in preparation for histological examination.

### 2.7 Procedure for Histology

The liver, kidney, uterus and ovary obtained from study animals were fixed in 10% formalin. They were routinely processed, using paraffin-wax embedding method. During sectioning, 5 µm thick sections were mounted on glass slides stained with Haematoxylin and Eosin. Histopathological assessment and photomicrography of the prepared slides was done. Photomicrographs were taken at ×100 and ×400 magnifications with a Digital Microscope, VJ-2005 DNMODEL BIO-MICROSCOPE®. using TS View CX Image® Software, File version 6.2.4.3. (Gunadi et al., 2020).

### 2.8 Physicochemical Characterization of Whole Seed Particles and the Lipid-Free *M. oleifera* Seed (MOSE)

#### 2.8.1 Particle Morphology

The surface morphology of particles of MOSE that was previously freed from the potentially toxic lipid portion (lipid-free seedcake) as well as whole seed powder (lipid-rich) was observed by scanning electron microscopy (Hitachi S-4000 microscope) according to an earlier method (Nnamani et al., 2014; Attah et al., 2020). In brief, samples diluted with double-distilled water were deposited on film-coated copper grids and the samples were allowed an overnight drying at room temperature. The dried samples were visualized under the microscope and images of the particle microstructure were captured.

#### 2.8.2 Particle Size and Polydispersity Index

The mean diameter and polydispersity index of the MOSE particles powder was measured using a Zetasizer Nano-ZS (Malvern Instruments, Worceshtire, United Kingdom) equipped with a 10 mW He–Ne laser employing the wavelength of 633 nm and a backscattering angle of 173° at 25°C.

#### 2.8.3 Zeta Potential Analysis

The zeta potential of the powdered MOSE particles was determined via electrophoreticmobility measurements using a Zetasizer Nano-ZS (Malvern Instruments, Worceshtire, United Kingdom). The zeta potentialwas calculated applying the Helmholtz–Smoluchowski equation (*n* = 3).

### 2.9 Mineral Composition and Preliminary Peptidomic Analysis of MOSE

The mineral elemental composition of MOSE including calcium (Ca), magnesium (Mg), potassium (K), iron (Fe), zinc (Zn), manganese (Mn) and copper (Cu) as well as heavy metals such as lead (Pb), cadmium (Cd), cobalt (Co), chromium (Cr) and nickel (Ni) were determined following the method described by the Association of Official Analytical Chemists (AOAC, 1990). All the determinations were carried out in triplicates. Briefly, after sample dehydration for 24 h at 105°C, 2 g each were digested with 8 ml of concentrated nitric acid, 2 mL concentrated sulphuric acid, and 2 mL hydrogen peroxide. This was followed by heat treatment for 4 h at 70°C and cooled; 20 m of distilled water was added, and further digestion was achieved by heat treatment in the presence of nitric acid and sulphuric acid. Drops of concentrated nitric acid was added until the complete oxidation of the organic matter assumed to have been reached when no further darkening of the solution occurs followed by a clear yellow colour solution on persistent heat application. Resulting mixture was cooled, insoluble solids filtered out with Whatman No. 42 filter paper and then transferred to suitable containers. The pH of the solutions was determined while the digested samples were read on Buck Scientific’s Atomic Absorption Spectrophotometer (AAS: Model 210/211 VGP, United States). The values of calcium, magnesium and potassium have been reported in percentage while iron, zinc, manganese, copper and the analyzed heavy metals have been presented in mg/kg of the dry weight of MOSE (Diyaolu et al., 2021).

The pre-purified aqueous extract of MOSE was preliminarily analyzed for isotopically resolved groups of peptide peaks performed on an ABSciex 4800 reflector TOF/TOF (time-of-flight) analyzer (Framingham, MA). Approximately 0.5 μL of the peptide-rich extract was mixed with 3 μL of saturated alpha-cyano-hydroxylcinnamic acid (matrix) prepared in 50% double distilled water (DDW), 50% acetonitrile, 0.1% trifluoroacetic acid (v/v/v) purchased from Sigma-Aldrich, St Louis, Missouri. This was spotted on the MALDI target plate and analyzed after allowing spot to dry in the dark. Acquired spectra were processed using the Data Explorer software v. 4.9 (Koehbach et al., 2013). To determine the general chemical nature of aqueous extracts of MOSE, the RPHPLC fingerprint of the extract was done. The lyophilized extract was reconstituted in double distilled water and the solution was filtered using further syringe filter with a pore size of 0.45 mm followed by analytical RP-HPLC analysis on a Dionex Ultimate 3000 HPLC (Thermo Fisher Scientific, Waltham, MA), applying a flow rate of 1 mL/min, 2% gradient in a 60 min run time.

### 2.10 Statistical Analysis

Values were expressed as mean ± standard error of mean and as percentage. Data were analyzed using one-way analysis of variance and Student’s *t*-test where applicable. *p* ≤ 0.05 was taken as the level of significance while Bonferroni’s post hoc multiple comparison test at 95% confidence Interval of difference was used.

## 3 Results

**FIGURE 1:**
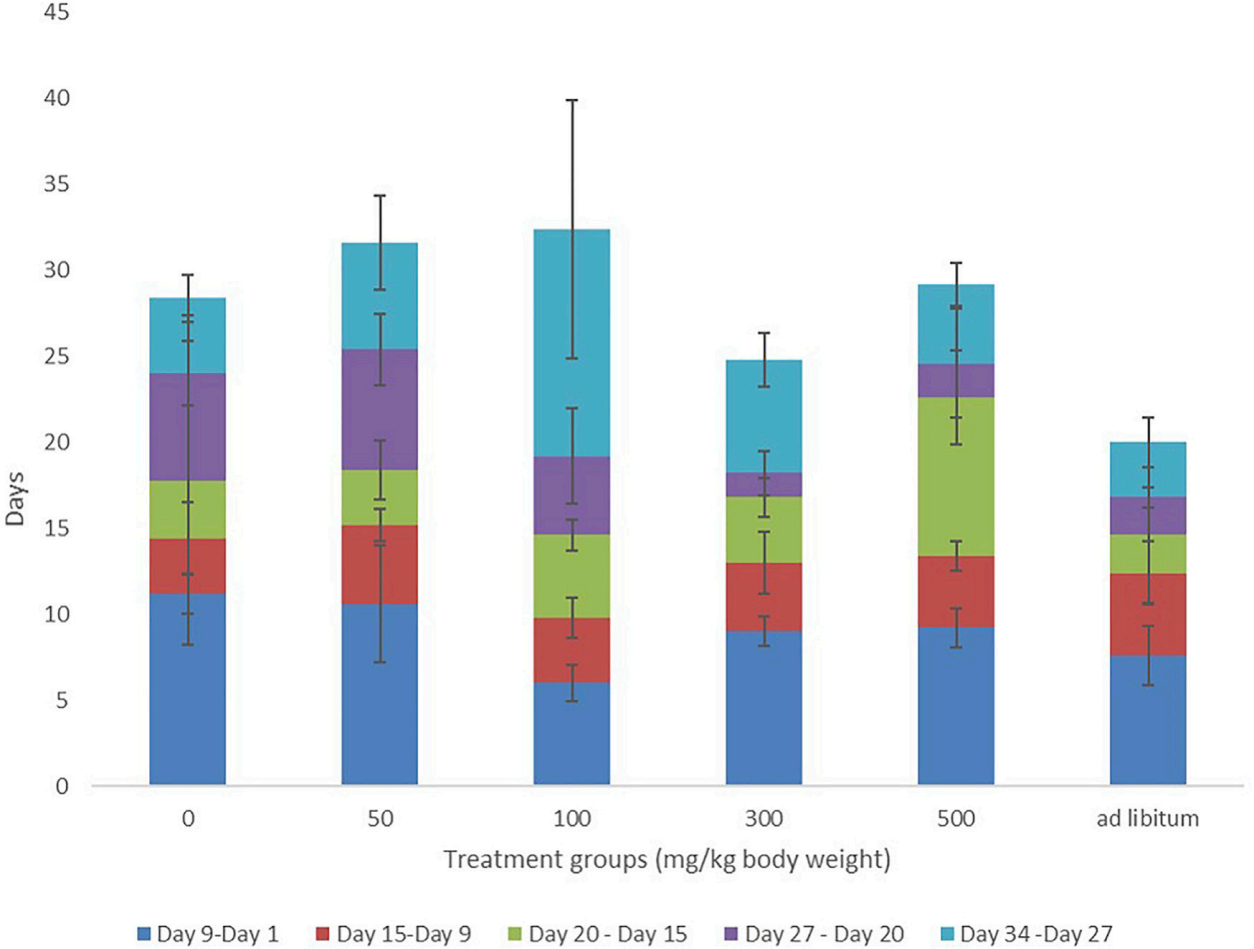
Weight variations of Dams over the period of administration of MOSE.

**FIGURE 2:**
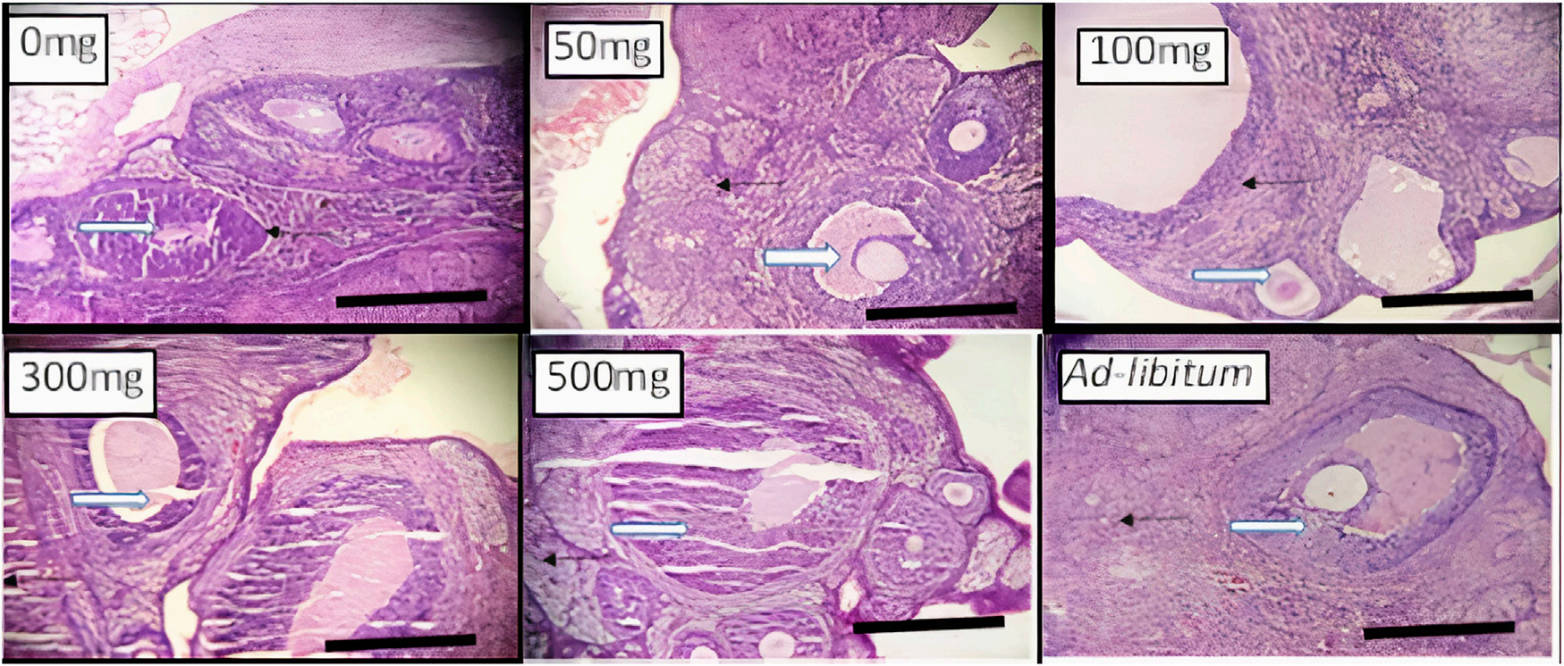
Effects of *Moringa oleifera* Aqueous Seed Extracts on Ovarian tissue of Dams; H&E, X100, scale bar = 50 μm. Photomicrograph of an ovarian sections showing normal, developing follicles (white arrow) within the cortex. Moderate infiltration of inflammatory cells is seen in stroma (slender arrow) of 0 mg only. The stroma (slender arrows) appears normal in all other groups.

**FIGURE 3:**
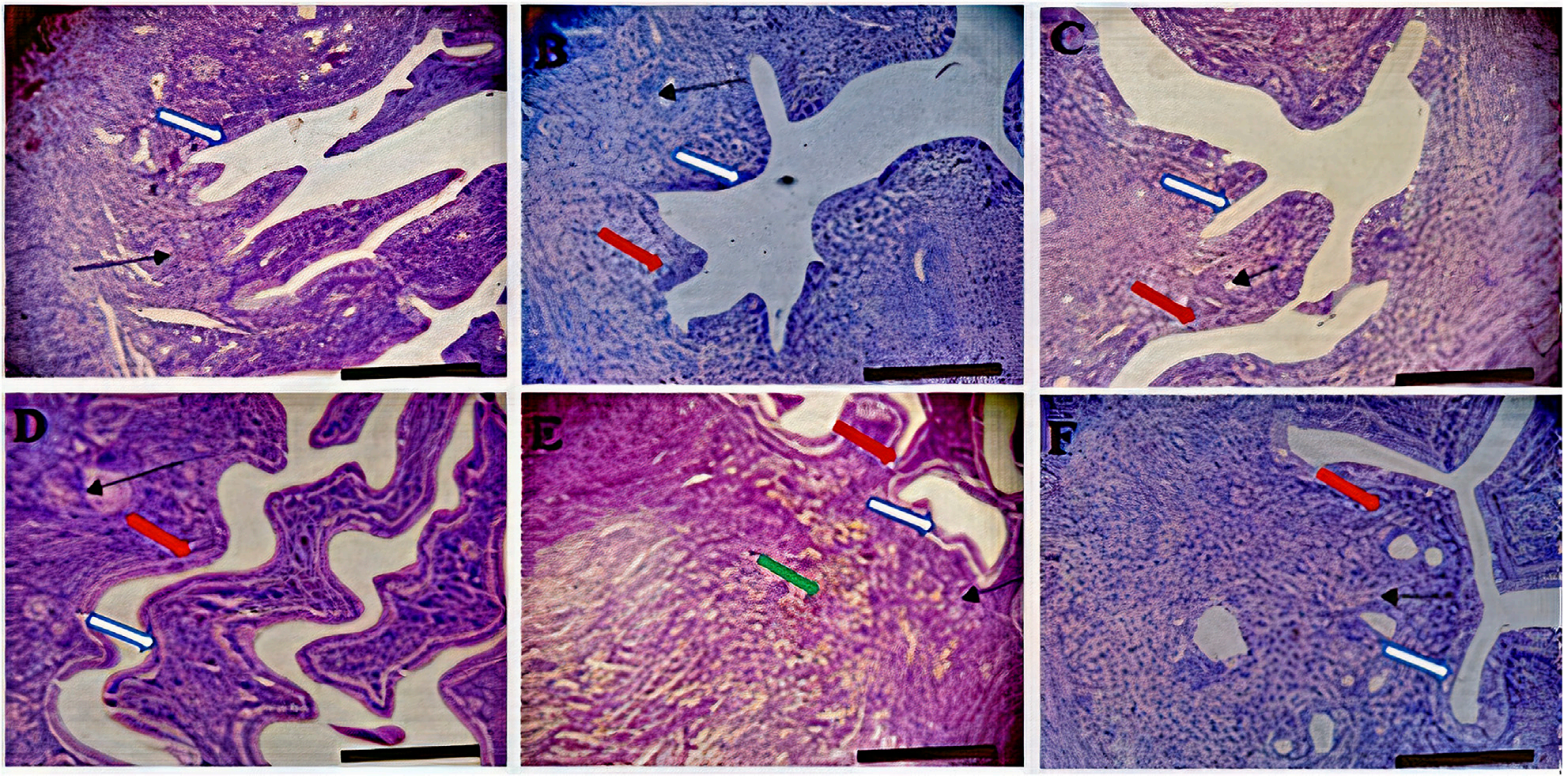
Effects of *Moringa oleifera* Aqueous Seed Extracts on Uterine Tissue of Dams; H&E, X100, scale bar = 50 μm. **(A)**—0 mg: Photomicrograph of uterus showing normal epithelial layer (white arrow) and normal endometrial glands (slender arrow). The lumen is normal. **(B)**—50 mg: Endometrial layer with normal epithelial layer (white arrow) and normal endometrial glands (slender arrow). The endometrium is infiltrated by inflammatory cells including lymphocytes and polymorphonuclear cells (red arrow). **(C)**—100 mg: Endometrium with normal epithelial layer (white arrow) and normal endometrial glands (slender arrow). The endometrium is infiltrated by inflammatory cells including lymphocytes and polymorphonuclear cells (red arrow). **(D)**—300 mg: Endometrial layer with normal epithelial layer (white arrow) and proliferated endometrial cells and glands (slender arrow). The endometrium is very mildly infiltrated by inflammatory cells (red arrow) including lymphocytes and polymorphonuclear cells. **(E)**—500 mg: Endometrial layer with normal epithelial layer (white arrow) moderately proliferated endometrial cells and glands (slender arrow) with mild infiltration of inflammatory cells (red arrow). Mild haemorrhage seen (green arrow). **(F)**—*Ad-libitum*: Endometrial tissue with normal glandular cells (slender arrow) and moderate infiltration of inflammatory cells (red arrow). The epithelial linings are normal (white arrow).

**FIGURE 4:**
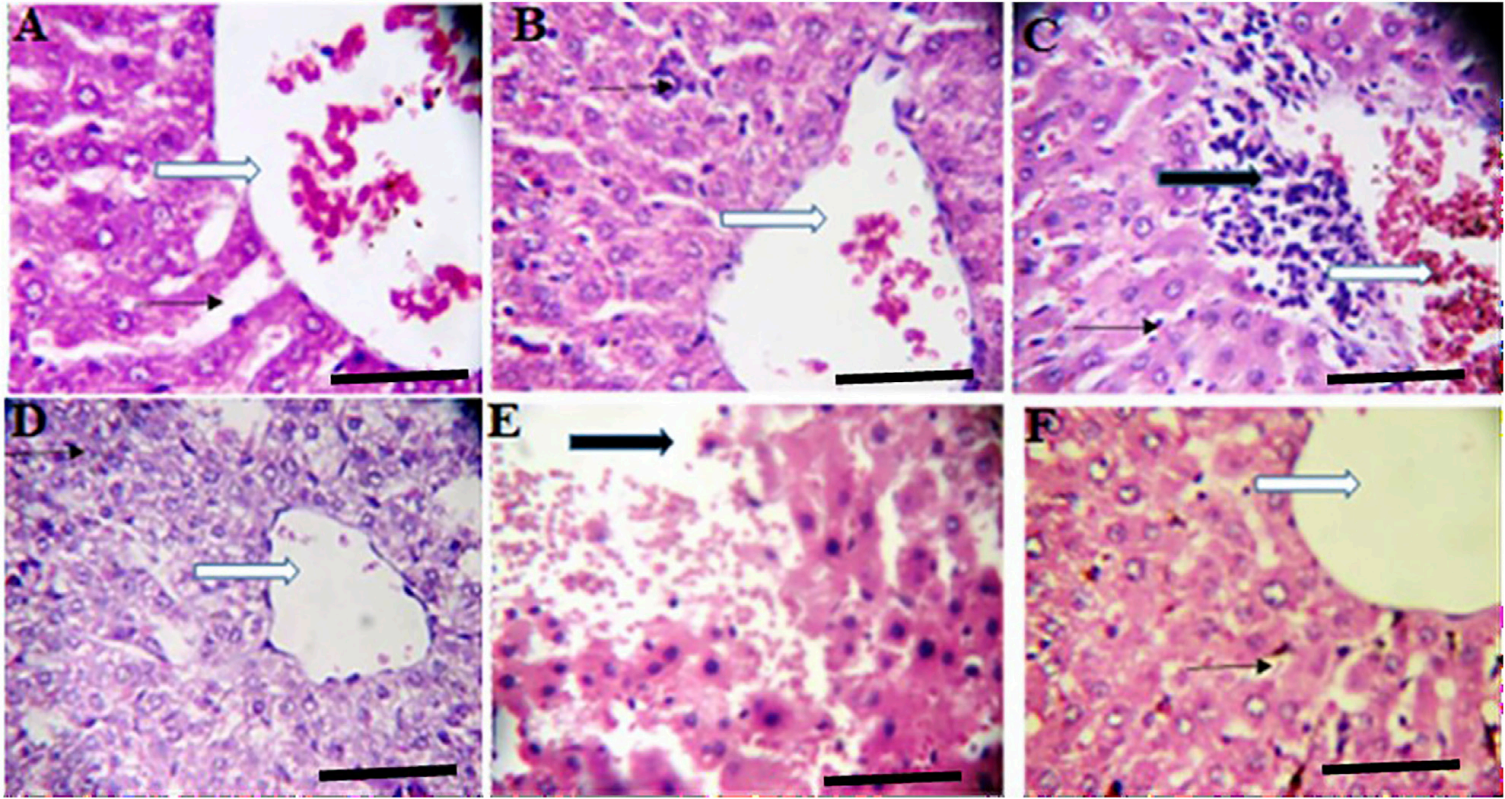
Effects of *Moringa oleifera* Aqueous Seed Extracts on Liver of Dams; H&E, X400, scale bars = 50 μm. **(A)**—0 mg: Photomicrograph of normal liver section showing normal architecture, the central vessel appears normal (white arrow), the sinusoids (slender arrow) appear normal there is no infiltration. The morphology of the hepatocytes appear normal and no pathological leision is seen. **(B)**—50 mg: Normal liver architecture, the central venule appear normal (white arrow), no vascular congestion seen, the sinusoids show mild infiltration of inflammatory cells (slender arrow), the morphology of the hepatocytes appear normal. **(C)**—100 mg: Moderately normal liver architecture with normal central venule (white arrow), there are some focal areas of mild diffusion of red cells infiltrating the liver parenchyma and sinusoids (slender arrow). There is periportal infiltration (black arrow). The morphology of the hepatocytes appear normal. **(D)**—300 mg: Poor liver architecture. The central venule (white arrow) appear normal, no vascular congestion seen, the sinusoids appear mildly infiltrated (slender arrow). The morphology of the hepatocytes appears normal. **(E)**—500 mg: Poor architecture with loss of liver plate and diffused red cells (black arrow). There are hepatocytes with hyperchromic nuclei (blue arrow), others appear normal. **(F)**—*Ad-libitum:* Normal liver architecture, the central venule (white arrow). No vascular congestion is seen and the sinusoids appear normal (slender arrow). The morphology of the hepatocytes appear normal.

**FIGURE 5:**
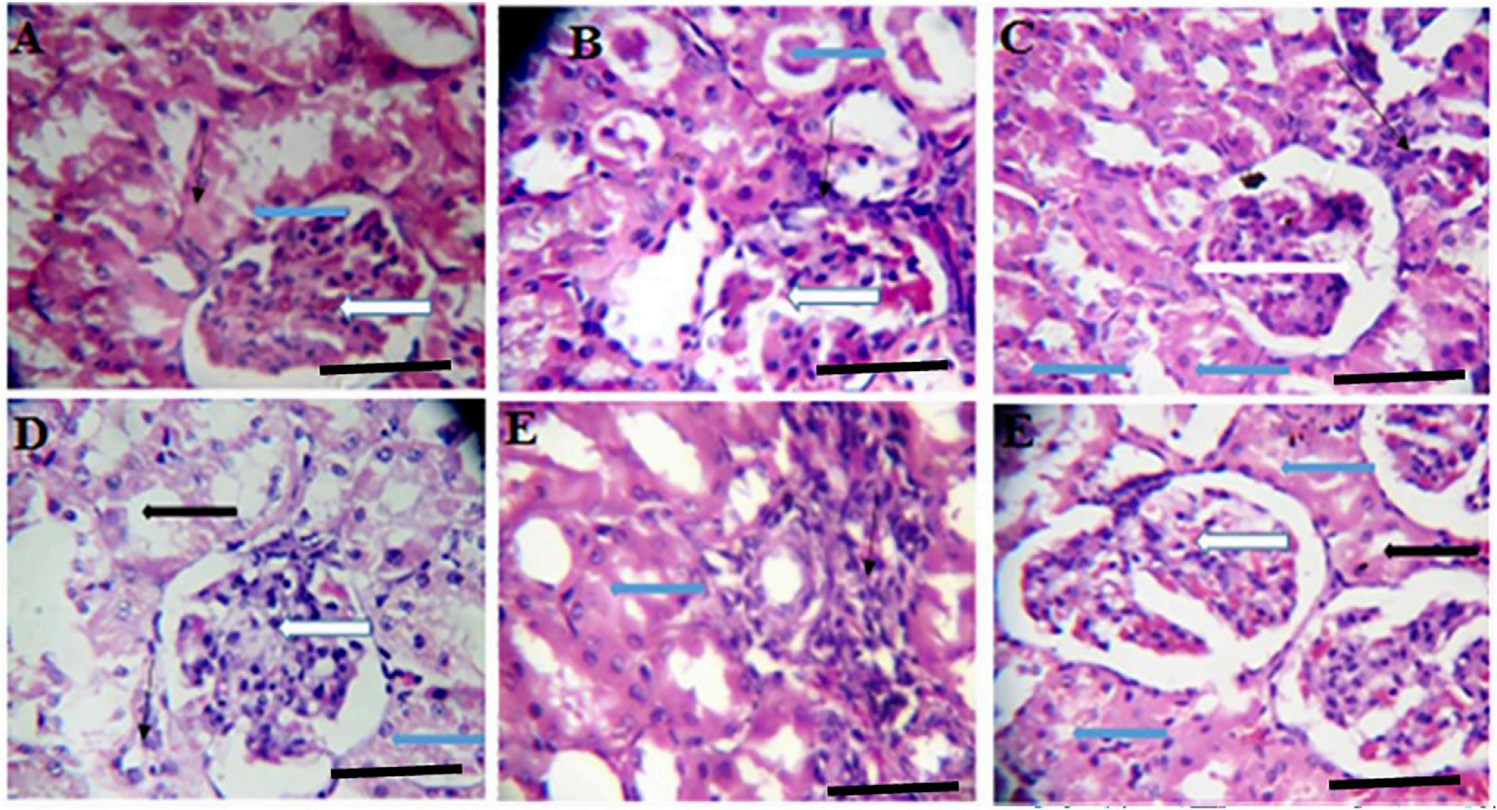
Effects of *Moringa oleifera* Aqueous Seed Extracts on the Kidney of Dams; H&E, X400, scale bars = 50 μm. **(A)**—0 mg: photomicrograph of normal kidney section showing normal renal cortex with normal glomeruli (white arrow) containing normal mesangial cells and capsular spaces. The renal tubules shows normal epithelial distribution and appear normal (blue arrow), the interstitial spaces (slender arrow) are not infiltrated. **(B)**—50 mg: Some glomeruli with mild mesangial cells hyperplasia (white arrow). The renal tubules show mild tubular necrosis and eosinophilic cast (black arrow) within their lumen. The interstitial spaces are midly infiltrated (slender arrow). **(C)**—100 mg: Normal glomeruli containing normal mesangial cells and capsular spaces (white arrow). The renal tubules show moderate tubular necrosis with loss of brush border (blue arrow). There is very mild infiltration of interstitial spaces (slender arrow). **(D)**—300 mg: Normal glomeruli containing normal mesangial cells and capsular spaces (white arrow). The renal tubules shows moderate tubular necrosis with loss of brush border (blue arrow) while other tubules are intact (black arrow). The interstitial spaces appear normal (slender arrow). **(E)**—500 mg: Tubules with moderate tubular necrosis (blue arrow). The interstitial spaces show scanty infiltration (slender arrow). **(F)**—*Ad-libitum*: Normal glomeruli containing normal mesangial cells and capsular spaces (white arrow). There is mild tubular necrosis (blue arrow) while other renal tubules appear normal (black arrow).

**FIGURE 6:**
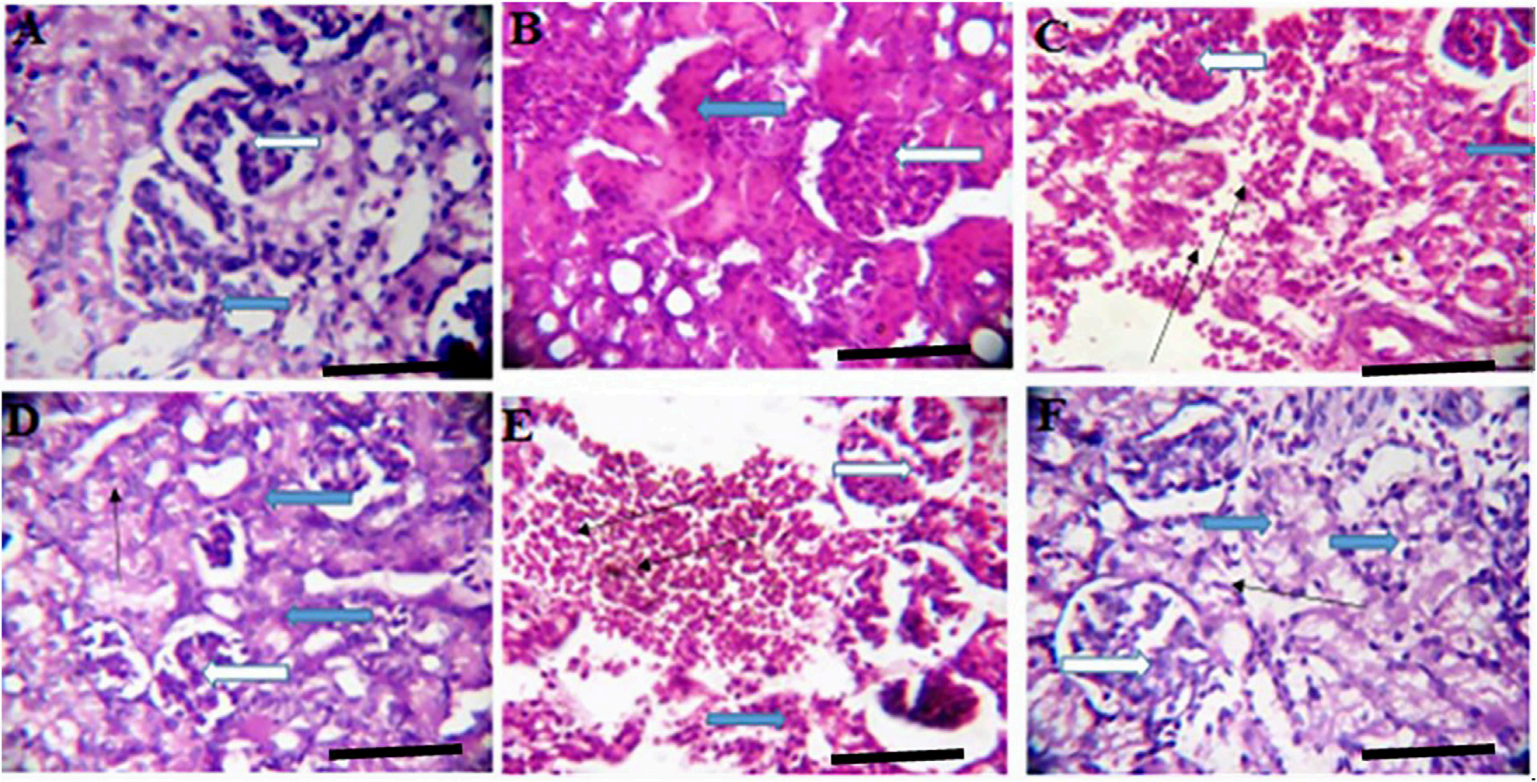
Effects of *Moringa oleifer*a Aqueous Seed Extracts on the Kidney of Pup; H&E, X400, scale bars = 50 μm. **(A)**—0 mg: Photomicrograph of normal kidney section showing normal multiple renal cortex with normal glomeruli (white arrow) containing normal mesangial cells and capsular spaces. The interstitial spaces show mild infiltration (slender arrow). **(B)**—50 mg: Numerous normal glomeruli within the renal cortex (white arrow). The renal tubules show attenuation and some tubules appear collapsed with lack of luminar spaces (black arrow). The interstitial spaces appear normal (slender arrow). **(C)**—100 mg: Numerous normal glomeruli within the renal cortex (white arrow). Some tubules are collapsed with diminished lumen (blue arrow) while others appear normal. The interstitial space is moderately widened and infiltrated by red cells (slender arrow) and few inflammatory cells. **(D)**—300 mg: Numerous compact glomeruli within the renal cortex (white arrow). The renal tubules shows acute tubular necrosis with loss of brush border (blue arrow). There are limiting lumen in the tubules as some tubules appear collapsed, the interstitial space is hardly defined (slender arrow). **(E)**—500 mg: Compact glomeruli within the renal cortex (white arrow). The renal parenchyma shows moderate fibrosis and tubules shows mild tubular necrosis (blue arrow). Some tubules are collapsed. There is vascular congestion seen in the interstitium (slender). The interstitial space is mildly infiltrated. **(F)**—*Ad-libitum*: Normal glomeruli containing normal mesangial cells and capsular spaces (white arrow). The renal tubules appear normal (blue arrow), the interstitial spaces appear normal and not infiltrated (slender arrow).

**FIGURE 7:**
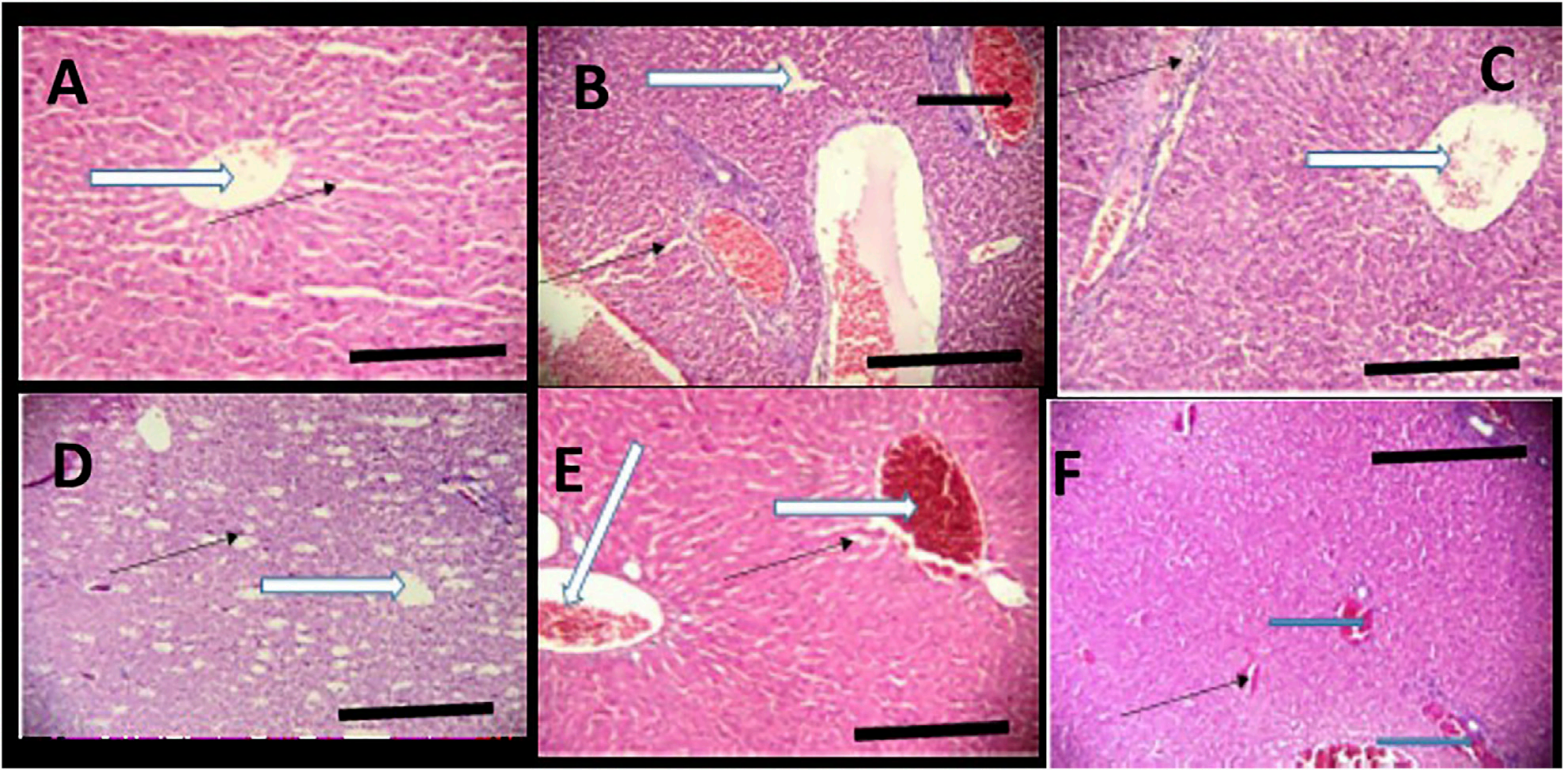
Effects of *Moringa oleifera* Lam. Aqueous Seed Extracts on the Liver of Pup; H&E, X100, scale bars = 50 μm. **(A)**—0 mg: Photomicrograph of normal liver section showing normal architecture, the central vessel appear normal (white arrow), the sinusoids appear normal (slender arrow) and not infiltrated. No pathological leision seen. **(B)**—50 mg: Poor liver architecture. There is moderate vascular congestion seen (black arrow). The sinusoids show no infiltration of inflammatory cells (slender arrow). **(C)**—100 mg: Moderately normal liver architecture with normal central venule (white arrow). There is mild focal area of mild diffusion of red cells and inflammatory cells infiltrating the liver parenchyma and sinusoids (slender arrow). **(D)**—300 mg: Central venule (white arrow) without congestion. The sinusoids arewide and mildly infiltrated (slender arrow). **(E)**—500 mg: Poor liver architecture. The venule and portal vein are congested (white arrow), the sinusoids show moderate dilatation with mild infiltration of inflammatory cells, (slender arrow). **(F)**—*Ad-libitum*: Normal liver architecture. There is mild vascular congestion seen (blue arrow), the sinusoids appear normal with no infiltration.

**FIGURE 8:**
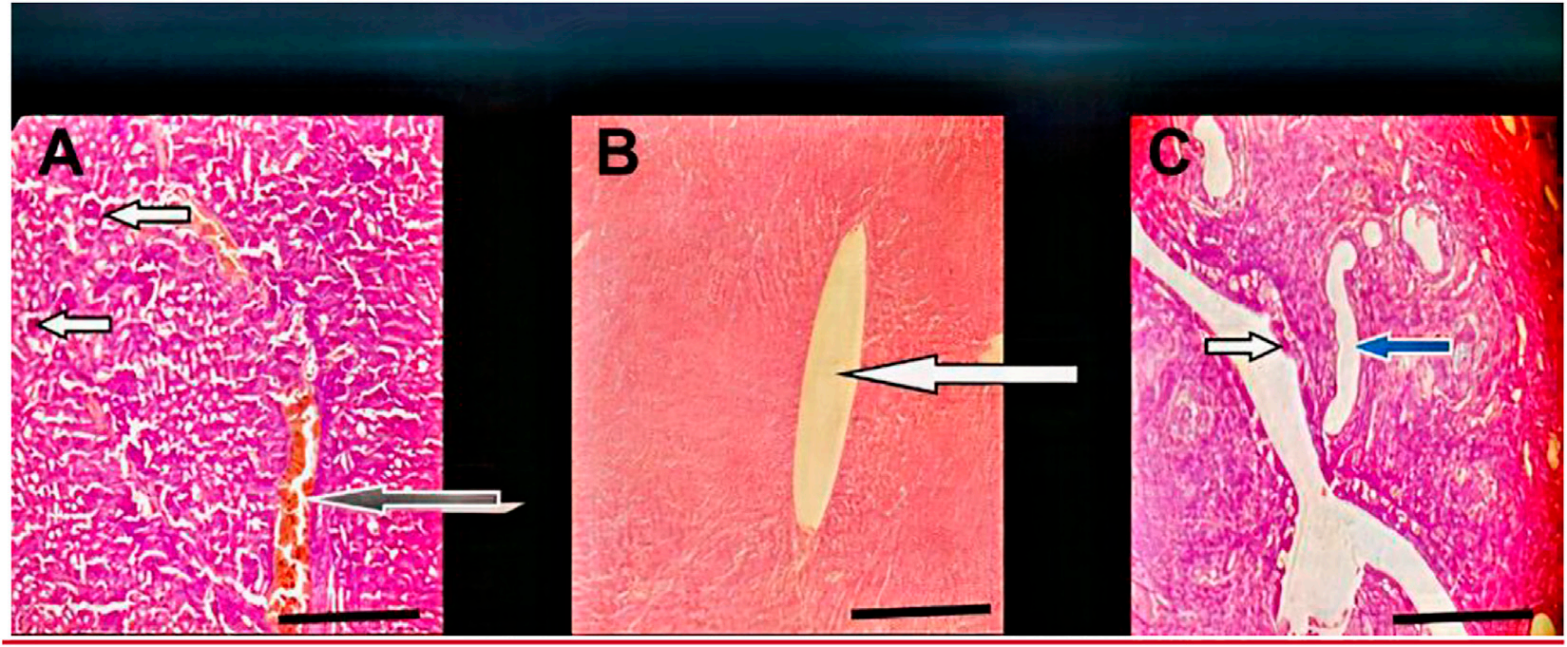
Effect of administration of 300 mg/kg body weight MOSE Cake freed of oil on some selected organs of Wistar rats; H&E, X100, scale bar = 50 μm. **(A)** Photomicrographs of kidney sections showing moderately normal architecture, the renal cortex shows normal glomeruli with normal capsular spaces and mesengial cells (white arrow), most the renal tubules appear normal (blue arrow), however, few area s with collapsed tubules are noted (red arrow). the interstitial spaces appear normal and not infiltrated (slender arrow). **(B)** Moderate architecture of the central venules and portal tracts are not congested (white arrows), the sinusoids show scanty infiltration of inflammatory cells (slender arrow). Hepatocytes show normal morphology (blue arrow). **(C)** Normal uterus, the epithelium (spanned) of the uterine mucosa layer (endometrium) appears normal (white arrow), the myometrium and perimetrium appear normal, the lumen is clean (red arrow), the uterine glands show mild dilatation (blue arrow) (blue arrow).

**FIGURE 9:**
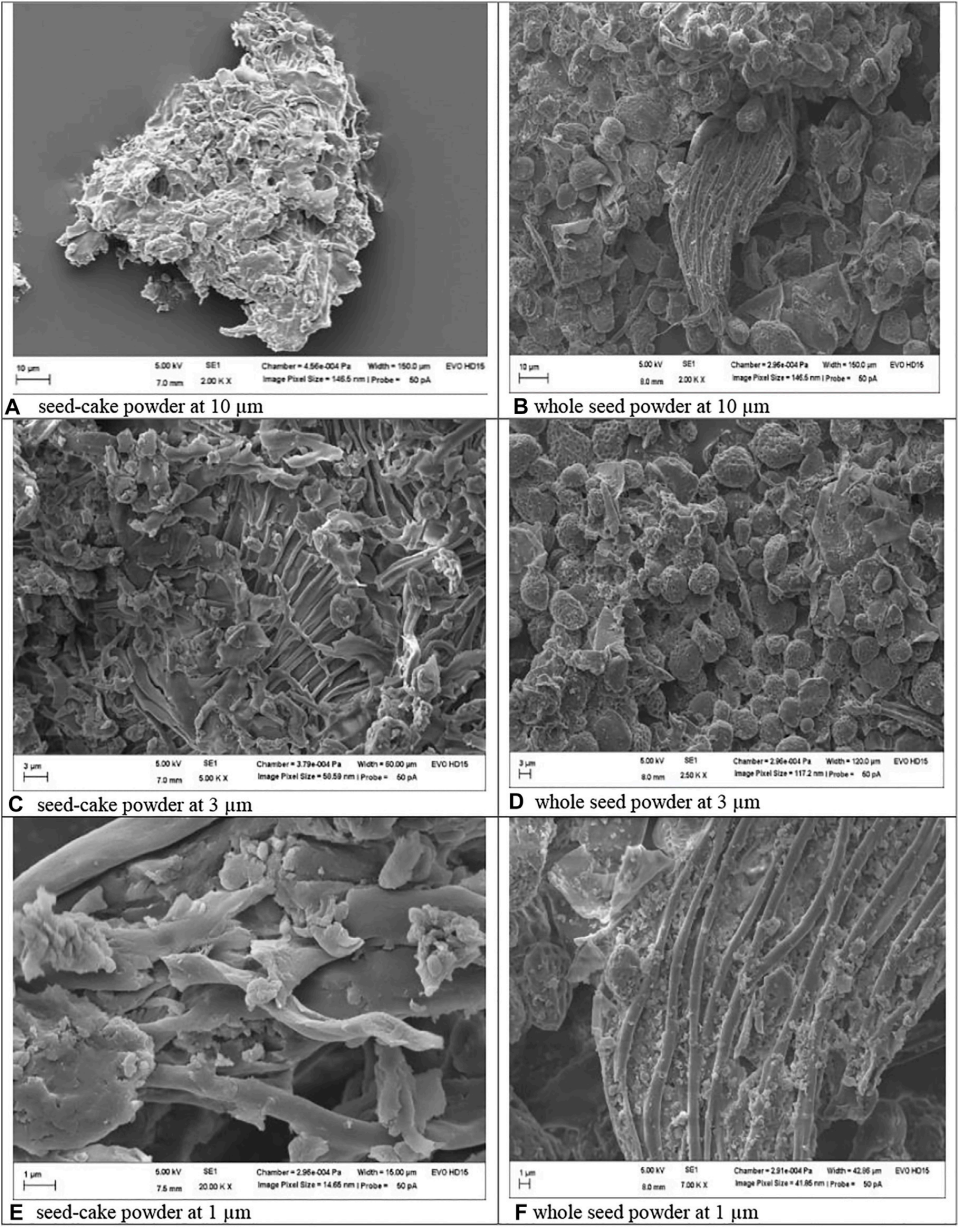
Morphology of MOSE as seed-cake powder (images ACE) and whole seed powder (images BDE). Mineral, heavy metal and RP-HPLC fingerprint of MOSE aqueous extract.

To obtain extracts of the lipid-rich MOSE containing some lipid component, an equal volume of distilled water and methanol was used to extract the powdered seeds resulting in a yield of 4.7% of the dry weight containing some lipophilic and hydrophilic constituents; as methanol is known to lyse cells to release its entire content. The 24 h hexane extraction resulted in removing 30% of lipids from MOSE. While seedcake extraction produced a yield of 3%. The administered extracts or MOSE-treated water has been carefully prepared considering both aspects of the traditional application of the seed in water treatment, as a food supplement and as a phytomedicine. The effect of the administration of MOSE on some indices in Dams has been presented in Table 1, while effect on pups is presented in Tables 2 and 3. Figure 1 presents the effect of MOSE on weight variation of Dams over the entire period of administration. Figures 2–7 presents the effects of lipid-rich MOSE aqueous extracts on selected key organs while Figure 8 demonstrates the effect of the extracts of lipid-free seedcake on selected organs. The physicochemical characters of the oil-rich and oil-free MOSE has been presented graphically in Figure 9.

**TABLE 1:** Effects of *Moringa oleifera* Lam. Aqueous Seed Extracts on Mating Index, Fertility index, Gestation Survival Index, Gestation Index and Gestation Length.

Groups	0 mg	50 mg	100 mg	300 mg	500 mg	Ad libitum
MI (%)	80 ± 1.80	80 ± 2.80	80 ± 1.30	80 ± 2.50	80 ± 3.50	80 ± 2.30
FI (%)	80 ± 1.40	80 ± 2.10	80 ± 2.50	80 ± 2.90	80 ± 2.80	80 ± 3.80
GSI (%)	100 ± 1.20	100 ± 3.30	100 ± 2.80	100 ± 3.20	100 ± 3.30	100 ± 2.50
GI (%)	100 ± 1.90	100 ± 1.80	100 ± 1.70	100 ± 3.00	100 ± 2.60	100 ± 3.10
GL (Days)	21 ± 1.30	21 ± 1.40	21 ± 1.10	20.8 ± 1.40	20.3 ± 1.0*	20.2 ± 1.20**

Values have been presented as percentages/Means ± SEM; *n* = 5. *p ≤ 0.05 and **p ≤ 0.01 compared with control. MI: Mating Index; FI: Fertility Index; GSI: Gestation Survival Index; GI: Gestation Index; GL: Gestation Length (20.3 and 20.2 may not be biologically significant due to the difference in mating time which was monitored once in a day).

**TABLE 2:** Effects of *Moringa oleifera* Lam. Aqueous seed extracts on morphometric indices of male pup.

Treatment	0 mg	50 mg	100 mg	300 mg	500 mg	*Ad-libitum*
Birth Weight (g)	5.5 ± 0.1	5.3 ± 0.1	5.3 ± 0.1	5.3 ± 0.3	5.5 ± 0.2	5.1 ± 0.1
ADI (mm/g)	1.5 ± 0.2	1.4 ± 0.1	2.1 ± 0.1	1.7 ± 0.2	1.5 ± 0.1	2.2 ± 0.1
HAR	0.90 ± 0.03	0.84 ± 0.03	0.85 ± 0.02	0.87 ± 0.03	0.91 ± 0.03	0.91 ± 0.01

ADI: Anogenital distance Index; HAR: head to Abdominal ratio; Values presented as mean ± SEM; *n* = 5.

**TABLE 3:** Effects of *Moringa oleifera* Lam. Aqueous seed extracts on female pup morphometric indices.

Treatment	0 mg	50 mg	100 mg	300 mg	500 mg	Ad-libitum
Birth Weight (g)	5.8 ± 0.1	4.3 ± 0.4*	4.7 ± 0.2*	4.8 ± 0.2	5.4 ± 0.2	5.5 ± 0.4
ADI (mm/g)	0.7 ± 0.1	0.9 ± 0.1	1.0 ± 0.1	0.9 ± 0.1	0.9 ± 0.1	1.2 ± 0.1**
HAR	0.77 ± 0.04	0.89 ± 0.02	0.86 ± 0.04	0.89 ± 0.04	0.94 ± 0.02	0.87 ± 0.02

Values presented as mean ± SEM; *n* = 5. *p ≤ 0.05, **p ≤ 0.01 compared with control. ADI: Anogenital distance Index; HAR: head to Abdominal ratio.

### 3.1 Effects of *Moringa oleifera* Lam. Aqueous Seed Extracts on Body Weight

Throughout the 2 weeks of administration (pretreatment period), there were no significant differences in the weight gained by the test groups compared with control. 50 and 100 mg groups had higher weight gain than control but this was not statistically significant. The weight gained by 500 mg and *ad-libitum* groups was significantly lower than that of the 50 mg group (Figure 1). Although the 50 mg, 100 mg, and 300 mg groups had increased weight gain during the post-treatment period, these changes were not significant compared with weight gain during the pre-treatment period. Likewise, 500 mg and *ad-libitum groups* had reduced weight gain during the post-treatment period but these differences were not statistically significant.

### 3.2 Effects of *Moringa oleifera* Lam. Aqueous Seed Extracts on Frequency of Estrous Phases

The length of estrous cycle during the pre-treatment phase and post-treatment phase did not change significantly in all groups. There were no significant differences in frequency of post-treatment proestrus, estrus and diestrus in all treated groups compared with the corresponding pre-treatment phases. The frequency of post-treatment metestrus in the 50 mg group was however reduced compared with the pre-treatment phase (*p* ≤ 0.05)**.**

### 3.3 Effects of *Moringa oleifera* Lam. Aqueous Seed Extracts on Mating Index, Fertility Index, Gestation Survival Index, Gestation Index and Gestation Length

Mating index, fertility index and gestational survival index were not different among all groups (Table 1). Gestation length was significantly reduced in the 500 mg and *ad-libitum* groups compared with control.

### 3.4 Effects of *Moringa oleifera* Lam. Aqueous Seed Extracts on Litter Size and Sex Ratio

Sex ratio in the test groups, which ranged from 1.0–5.0 was not significantly different from that of control (3.0). Litter size of 6–7 was documented for the test groups which has not shown any significant difference from the control group (litter size = 7).

### 3.5 Effects of *Moringa oleifera* Lam. Aqueous Seed Extracts on Pup Morphometric Indices

There were no significant differences in the birth weight, anogenital distance index and head to abdominal ratio of male pups belonging to MOSE-treated groups compared with control group (Table 2). Birth weights of female pups belonging to 50 mg, 100 mg groups were reduced compared with that of control pups. Anogenital distance index of the female pups from *ad-libitum* dams was increased significantly compared with the anogenital distance index of the control group (*p* ≤ 0.01). No significant difference was seen in the female pup head to abdominal ratio of test groups as compared with control group (Table 3). The weight variation documented during the treatment period has been presented in Figure 1.

### 3.6 Effects of Lipid-Rich *Moringa oleifera* Lam. Aqueous Seed Extracts on Selected Key Organs

#### 3.6.1 Safety Evaluation of Lipid-free MOSE

Following the organ toxicity observed with the lipid-rich MOSE, we carried out a follow-up single dose (300 mg/kg body weight) *in vivo* experiment to fully validate previously reported *in vitro* findings and our hypothesis: that MOSE whole seed may be toxic and the separation of the lipid portion from the metabolite-rich seedcake will exclude the reported *in vitro* cytotoxicity/genotoxicity and the *in vivo* organ toxicity of MOSE documented in this study. The chosen dose of 300 mg/kg body weight was selected as it represents the median dose at which pathologies were observed during this *in vivo* study. Result from this experiment excluded the renal and hepatic pathologies observed with the lipid-containing MOSE extract. The liver, kidney and uterus studied showed normal architecture with no observable pathologies (Figure 8).

#### 3.6.2 Physicochemical Characterization of MOSE Particles

The shape and surface morphology of both lipid-rich (whole seed powder) and lipid-free (seedcake) MOSE particles revealed a smooth and super porous spongy appearance (Figure 9), as seed-cake (images ACE) or whole seed powder (images BDF). Lower measurements of 1 µm showed somewhat more pronounced tubular channels in the whole seed powder than in the seed cake, perhaps due to presence of lipids in the whole seed powder. The particle size of MOSE particles was 848.6 nm with polydispersity index (pdi) of 0.75 and zeta potential measurement of −12 mV compared to the larger-sized whole seed powder (8.88 µm) with more variation in size (0.89 pdi) and slightly lower zeta potential value of −10.50 mV. However, it is clear that MOSE particles (seed cake powder or whole seed powder) were stable and had numerous pores and/or openings. Obviously, MOSE particles from the seedcake (without lipid portion) existed at nanoscale (∼849 nm size) and can be said to be a nanomaterial. Nanomaterials have been shown to have more interesting properties than bulk materials (such as MOSE lipid-rich whole seed powder). This could explain why MOSE seedcake particles (without lipid portion) had lower reproductive toxicity than the larger particles from the whole seed particles containing oil portions. Hence, oil removal from MOSE results in a seedcake whose particle characteristics implies a safer reproductive effects than whole seed particles. Additionally, this favors the storage stability of MOSE particles of the seekcake over the whole seed powder.

**FIGURE 10:**
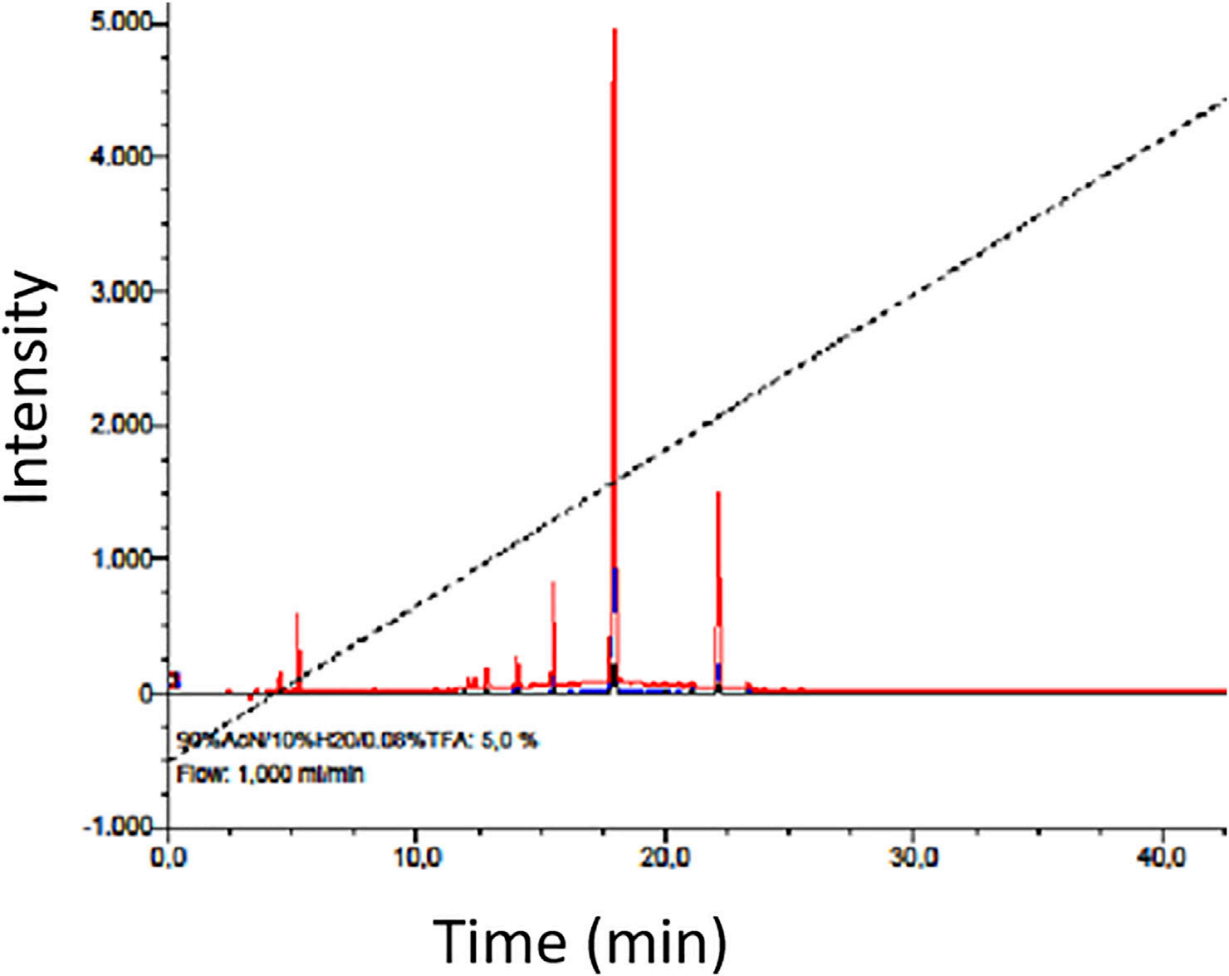
RP-HPLC chromatogram showing a general fingerprint and chemical nature of the prepurified aqueous extract of MOSE; mainly indicating early and late early eluting metabolites as expected which have been preliminarily analyzed by MALDI-TOF MS (Koehbach et al., 2013) to contain abundant peptide fragments (ranging from 1000 Da to 4.0 kDa within the mass window analyzed).

Results obtained from the mineral analysis indicate that MOSE is rich in essential minerals like Ca (0.077%), Mg (0.160%), K (0.30%), Mn (10.43 mg/kg), Cu (1.92 mg/kg), Fe (159 mg/kg), and Zn (20.92 mg/kg). The heavy metal component of MOSE including Pb (0.03 mg/kg), Cd (not detected), Co (not detected), Cr (0.021 mg/kg) and Ni (0.019 mg/kg) were all found to be lower than the limit set by WHO/FAO (Diyaolu et al., 2021). The RP-HPLC fingerprint of the aqueous extract of MOSE indicated early to late early elution of MOSE metabolites within 4–23 min retention time (Figure 10). The MALDI-TOF MS was further used to screen for peptide occurrence leading to the detection of abundant number of peptides ranging from 1.0—4.0 kDa within the mass window analyzed.

## 4 Discussion

Often ignored are the claims regarding the abortifacient potentials of some aerial parts of *Moringa oleifera* plant particularly the well valued fruit and potential toxicity of MOSE white kernels. Meanwhile, MOSE are now increasingly used as food additives, polluted water clarifier and as a phytomedicine without considering its potential toxicity. For instance, the *in vitro* cytotoxicity and genotoxicity of MOSE using the cell bioreporter model has been reported (Al-Anizi et al., 2014). To the best of our knowledge, this work represents the first report on the effect of MOSE aqueous extracts on fertility and pregnancy outcomes in rodents; although flocculant high molecular weight proteins have been characterized in Moringa seed kernel (Gassenschmidt et al., 1995; Ghebremichael et al., 2005; Dzuvor et al., 2022), we further report that MOSE seed kernel is rich in peptides and proteins as indicated by the findings from protein proximate analysis and the preliminary MALDI TOF-guided peptide detection. These numerous peptides are likely hydrolysates of bigger proteins which have been released during the harsh solvent-based extraction process.

This study investigated the effects of aqueous-methanol extracts of MOSE on, body weight gain, estrous cycle pattern, mating index, fertility index, gestation survival index, gestation index, gestation length, litter size, sex ratio, pup birth morphometric indices and selected organ histology. Here, the aqueous-methanol crude extracts of oil-rich and oil-free MOSE were used for the assessment of reproductive toxicity in female rats.

Aqueous MOSE extracts did not adversely affect the pregnancy length and the estrus cycle of the animals. This result tends to support the use of MOSE and its abundant polypeptides as flocculent molecules. However, it is of note that many bioactive agents reported in MOSE have been described as uterotonic compounds (Gruber and O'Brien, 2011). The very mild *in vivo* uterine contractility activities of MOSE aqueous extracts may be associated with the low occurrence of these uterine toning compounds in the seeds. More so, beta-sitosterol (a uterotonic agent) present in MOSE has been reported to be poorly absorbed in the gut (Babu and Jayaraman, 2020). This reduces the physiological beta-sitosterol and by extension reduces the abortifacient potential of the seed.

Also, MOSE did not have any statistically significant effect on body weight of rats. However, there was a dose dependent decrease in weight gain and the lower doses seemed to cause an increased weight gain while the higher doses caused reduction in weight gain. Several reports have shown that many phytochemicals present in medicinal plants negatively impact weight gain in animals (Jamroz et al., 2009; Van Hul and Cani, 2019). For instance, phenolic compounds such as tannins, which are reported present in MOSE (Santos et al., 2005) are capable of inhibiting digestive enzymes (Barrett et al., 2018) affecting the availability of amino acids, fatty acids and monosaccharides for absorption thereby leading to decreased weight gain. Foods rich in tannin are considered to be of low nutritional value (Chung et al., 1998; Manzoor et al., 2020) and the addition of a high percentage of tannin to animal diet has been reported to reduce weight gain while addition of low tannin showed no such effect (Mkhize et al., 2018). The chemical markers for West African MOSE include proteins, oleic acid and flavan-3-ols, indicative of lower tannin content in MOSE (Fernandes et al., 2021). However, a high dosage of MOSE may lead to a cumulative effect and this may be one of the reasons why the animals that took higher doses had significant reduction in body weight gain compared with the 50 mg group. The question still remains, why was there an increase in body weight gain of the 50 mg group? MOSE is rich in carbohydrate, protein and fat and oil (Fernandes et al., 2021) and should naturally nourish the body. These rich dietary constituents, especially the fat content may be responsible for the increase in weight gain seen in the 50 mg group. However, as the dosage increases, so does the tannin content that naturally acts to inhibit digestive enzymes and prevents the availability of the end products of these rich dietary constituents.

The length of the estrous cycle was not affected by MOSE intake; however, the frequency of metestrus phase was reduced in the 50 mg group while the frequencies of other phases were not affected. Since the length of the cycle remained the same and reduction in metestrus frequency was not accompanied by alteration in the frequency of the other phases, MOSE did not seem to have any adverse effect on the length or regularity of estrous cycle. More so, metestrus is a transient phase immediately following ovulation in rats and this period may be very short or even absent (Ajayi and Akhigbe, 2020).

Histology of the ovary suggests that MOSE prevented pre-ovulatory inflammation. The infiltration in the ovary of the control rat was not a pathological reaction because proestrus seems to be associated with mast cell degranulation resulting in inflammation (Xu et al., 2020) that may be needed for the initiation of ovulation or estrus. Results from this study corroborates the results of other studies that reported the anti-inflammatory effects of *Moringa oleifera* seed extract (Xiong et al., 2020). This result also suggests that an increase in the frequency of metestrus was the outcome of prolonged proestrus. Though the alteration in frequencies of proestrus was not significant during the 2 weeks of administration, the deviation pattern of the cycle suggests that chronic intake could cause a significant effect which may eventually alter the pattern of the cycle. Contrary to the anti-inflammatory activity exerted by MOSE in the ovaries, the uterine and liver tissues of all test groups had infiltration of inflammatory cells. Why this is so being not understood. Administration of MOSE induced renal toxicity as tubular necrosis was observed in all the groups that took it. Pathological changes were also observed in the liver of rodents investigated. These *in vivo* findings suggest that the lipid-rich MOSE extract may be toxic to the liver and kidney. This contradicts several reports in local journals regarding the hepato-protective and nephroprotective effects of MOSE but is in agreement with well investigated *in vitro* reports by Al-Anizi et al. (Al-Anizi et al, 2014). These opposing reports may be associated with the part of the plant used for the assay, geographical variations or other extant factors. In this experiment, only the aqueous extracts of the husked seed (called kernel) of MOSE was used. This approach is most similar to how the seed is used in ethnomedicine, as food additive and as polluted water clarifier.

Fertility indices of the test animals in this study were not different from that of control. The result on litter size also suggests that implantation was not affected since the MOSE-treated groups had similar litter size as the control group. Gestation length appeared not to be significantly affected across all the experimental groups. Thus, all pups born to the MOSE-treated mothers were viable at birth and till sacrifice. This finding is in line with a report on the improvement of poultry egg characteristics without any effect on fertility when given MOSE (Ashour et al., 2020).

Dietary method of preconceptional selection of fetal sex relies on the ionic content of the female diet in humans (Stolkowski and Lorrain, 1980; Alhimaidi et al., 2021). MOSE is rich in potassium, calcium, magnesium and phosphorus and did not affect sex ratio in this work. Diets with a high ratio of sodium and potassium to calcium and magnesium favour the birth of male offspring while the low ratio of these ions favours the birth of female offspring (Alhimaidi et al., 2021). The MOSE used during this study are richer in calcium and potassium than in magnesium. The distribution of these ions in MOSE appear not to be in conformity with the classical pattern of ion distribution that has been suggested to lead to more male or female births.

The environment encountered *in-utero* by a developing fetus exerts a profound influence on physiological function and risk of disease in adult life (Calkins and Devaskar, 2011; Hsu and Tain, 2019). Anthropometry in humans and morphometry in animals could provide useful information for determining the risk of disease in adult life (Tuvemo et al., 1999). The male pup morphometry was not affected by MOSE treatment. The reduction in birth weight of the female pups belonging to the 50 and 100 mg groups suggests that maternal MOSE-treatment had a fetal programming effect on these offspring. The implication of this is that these pups may develop a range of diseases later in life. The female pups of the *ad-libitum* groups also showed a sign of masculinization as they had increased anogenital distance index. Alterations in the human anogenital distance were first seen in female infants that had congenital adrenal hyperplasia, and they were presented with longer anogenital distance than girls without the disorder (Hsieh et al., 2008). Anogenital distance measurement in humans is a non-invasive method of determining the degree of masculinization and androgen exposure during fetal life (Schwartz et al., 2019). *Moringa oleifera* seed extract was reported to exert an androgenic effect in a study (Obembe, 2019) and such effect during gestation may be associated with the masculinization of the female reproductive organs as well as the female brain.

Findings from this study indicate that the liver and kidney of pups belonging to the treated groups were programmed *in-utero* by maternal MOSE intake. This was evident in the varying degrees of pathologies seen in these tissues as early as 2 weeks’ post-natal life. This suggests that intake of MOSE during gestation may be toxic to fetal development. The effects exerted on the kidney and liver were not gender specific and did not seem to be dose-dependent. Result obtained from the heavy metal occurrence in MOSE which were all below standard limits set by the WHO/FAO suggests that the toxicity of lipid-rich MOSE used in this study was not associated with the occurrence of toxic metals. This further provides support for the work of Al-alnizi (Al-Anizi et al., 2014) regarding the cytotoxicity and genotoxicity of lipid-rich MOSE which was excluded when MOSE was freed of its lipid content and administered to the test rodents at a dose of 300 mg/kg body weight. Findings from this study are also consistent with a recent report on the toxicity of *Moringa stenopetala*, a close member of the genus Moringa, on the reproductive indices of rodents (Teshome et al., 2021).

Aside providing insight to the nature of metabolites present, the RP-HPLC fingerprint as well as the abundant peptide masses detected by the MALDI TOF peptidomics provide a useful link between the hydrophilic less toxic component of MOSE and the lypohpilic toxic component of MOSE and further validated the efficiency of the extraction of the two (i.e aqueous lipid-free and lipid-rich) components of MOSE used in this study; hence, the analytical RP-HPLC chromatogram of aqueous extract of the lipid-free MOSE indicated presence of hydrophilic compounds (with early retention times) as well as less hydrophilic metabolites (hydrophobic behaviour) as judged by the major signals observed. This result is consistent with a large body of literature on the characterization of hydrophilic and less hydrophilic compounds (including, phenolics, glycosides and alkaloids) from MOSE (Xiong et al., 2020; Dzuvor et al., 2022; Xiong et al., 2021). Together, these results provide starting point and could guide future further mechanistic studies.

The physicochemical characterization of MOSE particles demonstrated clearly that MOSE particles (seed cake powder or whole seed powder) were stable and had numerous pores and/or openings. These pores could possibly be responsible for the efficient occlusion, entrapment, adhesion or attachment of moieties to MOSE particles when used as medicine, nutraceutical, food and for water clarification. The particles were more amorphous in the seed-cake forms (images A, C and E) compared to the whole seed powder (images B, D and F) possibly due to the absence of lipids which in other words were responsible for the bigger size, more tubular appearance and possibility far less storage stability of the whole seed powder particles. Meanwhile, MOSE particles of the seekcake existed at the nanoscale (∼849 nm) and as a nanomaterial could be expected to have more attractive properties than the bulk whole seed powder. This could explain why the former had safer reproductive effects that the later. A link between the physicochemical characteristics of a compound or nanoparticle and its toxic manifestation has been reported and this suggest that physicochemical properties generally influence the toxic manifestations of these phytochemical compounds and nanomaterials (Price et al., 2009). It has been documented that the physicochemical properties of food are mainly responsible for the final quality of the product which could make it more or less toxic (Mat Lazim et al., 2021). Moreover, the analysis of these properties is crucial for design and quality control during food processing. Here we present MOSE as food and medicine thus supporting the need for the physicochemical characterisation of MOSE particles.

In the absence of the toxic lipid portion, our findings suggest that MOSE seedcake possess far more interesting particle characteristics suitable for optimal product development and efficient manufacturing and this lends support to the potential efficiency and effectiveness in the application of the seedcake at the interphase of food and medicine. Additionally, findings from the physicochemical characterization of MOSE suggest that the application of the lipid-free seedcake of MOSE at the interface of food and medicine will not only make it safer and healthier, but will extend the shelf life of the product.

## 5 Conclusion

It is concluded in this study that oral administration of lipid-rich *Moringa oleifera* seed (MOSE) did not produce any serious adverse effect on estrous cycle and fertility of female Wistar rats. However, female offspring of the dams showed evidence of intra-uterine growth restriction and masculinization. Documented in this study is intrauterine programming of hepatic and renal organs of the offspring of MOSE-treated dams and this effect seemed to be more profound on the female offspring. Furthermore, the kidney and liver tissues showed varying degrees of pathologies in both male and female offspring suggestive of toxicity. Interestingly, the observed toxicity was excluded when lipid-free MOSE was administered at a dose of 300 mg/kg body weight. At the interface of food and medicine, common anecdotal claims on the intake of MOSE for medicinal purposes may be beneficial, however, this is discouraged except after excluding the lipid portion. The addition of the nutrient-laden MOSE powder yet to be defatted as a nutritional food additive may be unsafe. We suggest the safe use of lipid free but peptide rich seedcake for nutritional fortification.

This work therefore supports the safe use of lipid free MOSE extract and polypeptides for achieving clean drinking water in economically deprived settings as previous *in vitro* and our first reported *in vivo* evidence supports the toxicity of the lipid-rich kernels. The hydrophobic constituents have been associated with observed cytotoxicity and genotoxicity, and should be removed prior to its use for water treatment, as Food additive or for formulation into an effective phytomedicine. For polluted water treatment, the lipid-free seed cake may equally be adsorbed unto sand particles that have been innovatively parked into a stainless metal filter tube for point-of-use in households as well as for small rural community application; however, a pilot study on this is suggested for proof-of-concept.

## Data Availability

The raw data supporting the conclusion of this article will be made available by the authors, without undue reservation.
